# Optimization of Process Parameters for Preparing Metallic Matrix Diamond Tool Bits by Microwave Pressureless Sintering Using Response Surface Methodology

**DOI:** 10.3390/ma11112185

**Published:** 2018-11-05

**Authors:** Li Yang, Liang Wang, Jiyun Gao, Shenghui Guo, Xiaolei Ye, Sivasankar Koppala, Tu Hu, Ming Hou, Longtao Hu

**Affiliations:** 1 State Key Laboratory of Complex Nonferrous Metal Resources Clean Utilization, Kunming University of Science and Technology, Kunming 650093, China; yanglikmust@163.com (L.Y.); pepsiva9@gmail.com (S.K.); hutu1219@126.com (T.H.); houmingkmust@163.com (M.H.); 2 State International Joint Research Center of Advanced Technology for Superhard Materials, Kunming University of Science and Technology, Kunming 650093, China; 3 National Local Joint Laboratory of Engineering Application of Microwave Energy and Equipment Technology, Kunming 650093, China; kmust654321@163.com; 4 Faculty of Metallurgical and Energy Engineering, Kunming University of Science and Technology, Kunming 650093, China; 5 Yibin Tianyuan Group Co., Ltd., Yibin 644004, China; wangliangkmust@163.com; 6 School of Chemistry and Environment, Yunnan Minzu University, Kunming 650093, China; jiyungao89@163.com

**Keywords:** response surface methodology, microwave, pressureless sintering, diamond tool bits, metallic matrix

## Abstract

The process of preparing metallic matrix diamond tool bits by microwave pressureless sintering (MPS) was exclusively studied in this paper. The effects of the sintering temperature, the cold pressure, and the holding time on the mechanical properties of the bit were determined by using the response surface methodology (RSM) with Box-Behnken Design (BBD). In addition, with RSM, the second-order polynomial equation of mechanical properties was obtained. The solutions were well matched with the experimental values. This indicates that major variations in mechanical properties of the sintered sample could be predicted by the models, which shows that the applied model is accurate. Conventional pressureless sintering (CPS) experiments were also conducted to make a comparison. The experimental results showed that the MPS can enhance the mechanical properties of sintered samples. A possible MPS mechanism is proposed in this work after analyzing all the experimental results.

## 1. Introduction

Metallic matrix diamond tools are widely used in machining, construction, and geological drilling industries due to their high processability, good wear resistance, high hardness, and high thermal conductivity [1,2,3]. Typically, metal matrix diamond tools contain abrasive particles and a metal matrix [4]. Generally, diamond tools can be prepared by conventional hot press sintering (CHPS) or conventional pressureless sintering (CPS) methods [5,6]. However, there are certain drawbacks when fabricating metallic matrix diamond tools by the CHPS or CPS methods. In the case of the CHPS method, although the sintering time is shortened due to the simultaneous application of the axial compressive force to the compact material during the sintering process, a large amount of expensive graphite mold will be consumed [6,7]. In the CPS method, the preparation strategy is highly inefficient due to longer working time needed to accommodate the alloying reaction [8]. The energy absorption modes of the CHPS and CPS methods depend on the heat transfer and heat radiation modes of the conventional resistance furnace from the outside to the inside, which is opposite to the direction in which the gas molecules leak. The direction of the contradiction will also hinder the evolution of the gas and the shrinkage of the microstructure when the sample is being densified [9]. To overcome the above major drawbacks, efficient new sintering methods for the preparation of metallic matrix diamond tools must be developed.

Microwave technology is widely accepted for its unique advantages such as rapid heating, overall heating, and internal heating [10,11]. Especially, since the 1950s, microwave energy has gained attention in the field of material preparation and many researchers have studied the microwave preparation technology of metallic materials [12,13]. Xu et al. reported that copper powder can melt rapidly in the microwave field [14]. Mondal et al. prepared a 90W-7Ni-3Fe alloy with finer microstructure, higher hardness, and flexural strength by microwave sintering than conventional sintering [15]. Ripley et al. successfully heat treated Fe, Ti, Zr, U, Cu, Al, and their alloys in the microwave field [16]. Based on the above reports, we decided to check whether microwave energy is useful for making metal matrix diamond tools. Actually, we have also done the exploration of microwave sintered diamond tools in the early stage and achieved some results [5,6,7]. Lastly, we tried to optimize the microwave sintering process by using the RSM.

RSM is used to optimize many variables that affect experimental results, which are derived from the Analysis of Variance (ANOVA) [17]. The experimental workload can be reduced by using this statistical design. Furthermore, the connection between the variable and the response variables can be estimated by the quadratic regression model [18]. In this work, RSM with a Box-Behnken Design (BBD) was used to optimize the sintering conditions of MPS to prepare metal matrix diamond tools and to obtain a regression quadratic mathematical model for evaluating individual and interaction effects of various parameters. It is reflected in the influence of each independent variables (sintering temperature, cold pressure, and holding time) on the response variables (relative density, flexural strength, and abrasive ratio). In addition, we have also conducted a comparative study of MPS and CPS after optimization. Scanning electron microscopy (SEM, JSM-5610LV, JEOL, Osaka, Japan) was used to detected the microstructure information of the samples. Furthermore, possible mechanisms are discussed to explain the merits of the MPS approach.

## 2. Materials and Methods

### 2.1. Materials and Equipment

**Figure 1:**
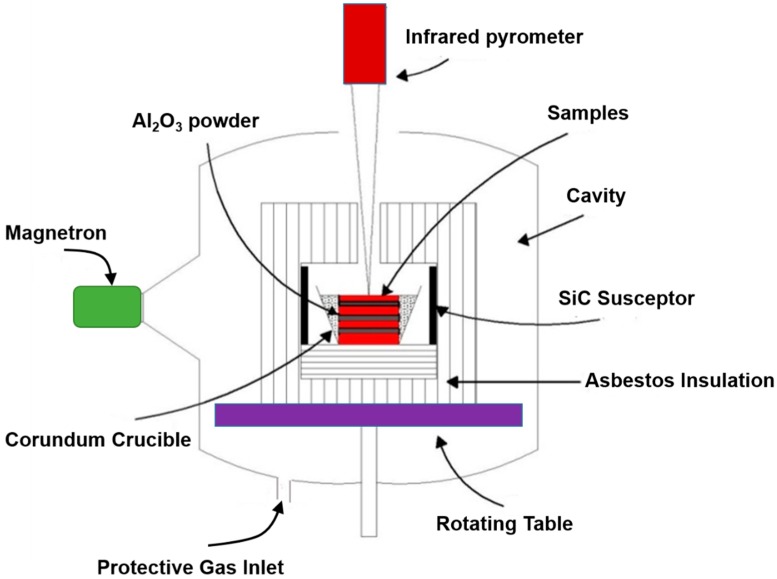
Cavity profile of microwave sintering furnace.

Henan Huanghe Whirlwind Co. Ltd. (Zhengzhou, China) provides diamond abrasive (99.98%, 35 mesh), GRIPM Advanced Materials Co., Ltd. (Beijing, China) provides copper powders (Cu, 99.99%, 200 mesh), iron powders (Fe, 99.99%, 200 mesh), cobalt powders (Co, 99.99%, 200 mesh), tin powders (Sn, 99.99%, 200 mesh), nickel powders (Ni, 99.99%, 200 mesh), and titanium powders (Ti, 99.99%, 200 mesh). A three-dimensional vortex mixer (MX-2) and uniaxial automatic hydraulic press of 100T capacity (CP100) were purchased from Zhengzhou Golden Highway Co., Ltd. (Zhengzhou, China). The MPS process was carried out in a home-made microwave furnace (6 kW, 2.45 GHz), which is shown in Figure 1. It should be noted that this is a multimode microwave furnace. In general, a multimode heater couples a microwave of a certain power to a sealed metal box and then supplies it to a heating cavity. This mode is conducive for the design of large cavity microwave ovens suitable for industrial promotion. In the later stage of sintering, the ability to respond to microwaves is reduced due to gradual densification of the sample [12,14]. Therefore, the addition of SiC susceptor facilitates the heating of metal materials by microwaves. The green sample was placed in a corundum crucible on a rotating table. A protective gas was then introduced. The rotating table is turned on and the microwave emitting device is activated to uniformly apply the microwave to the sample. The temperature of the sample was detected by an infrared temperature measuring device. In addition, corundum is wrapped in asbestos insulation while filling the gap between the sample and corundum with Al_2_O_3_ powder for better insulation. As a comparative test, the CPS was carried out by heating in a vacuum sintering furnace with high purity Ar (99.99%).

The choice of the metal matrix is also an important process in the preparation of metal-based diamond tools. It should be pointed out that the good formability of Cu powder is favorable for cold press forming and sintering and Fe is widely used as an inexpensive metal and has good wettability with diamonds. The addition of Co can increase the flexural strength of the metal matrix while Ni can improve the wear resistance and toughness of the metal matrix. Sn is used as a liquid phase element to facilitate shrinkage densification of the metal matrix during sintering. Ti can form a metallurgical bond with diamond to improve the holding force of the metal matrix on the diamond. Therefore, the formulation design shown in Table 1 was selected. Metal powders and diamond were mixed for 1 h. The mixed powder was compacted to green compacts at a pressure of 200 MPa.

Then the MPS and CPS processes were performed. The heating rate from room temperature to 300 °C was 30 °C/min. The heating rate from 300 °C to 600 °C was 20 °C/min. Lastly, the temperature was raised to the desired temperature at a heating rate of 10 °C/min and kept for a certain period of time. Removing the sample from the furnace cooled to 180 °C after a certain time of incubation.

### 2.2. Characterization

The microstructure and the crystal phase of the sintered sample were observed by scanning electron microscopy (SEM) after polishing the sample. The density of the sintered sample was tested by the Archimedes drainage method and the relative density was calculated. The flexural strength of the sintered samples was measured by the hydraulic universal testing machine (AG-10TA, Shimadzu, Japan). The flexural strength was calculated, according to Equation (1) [19].

(1) $$\;\left(R_{tr}=\frac{3fL}{2bh^{2}}\right)\;$$

The flexural strength (MPa) is represented by *R_tr_*. The force required for fracture (N) is represented by *f*. The span (mm) is represented by *L*. The width and thickness of the sample were represented by *b* and *h*, respectively. The abrasive ratio was tested with SiC as the grinding material [7].

## 3. Experimental Design

RSM with BBD was used to optimize the sintering conditions (Sintering temperature, Cold pressure, Holding time) of MPS. RSM was used to compare the interactions between various variables after they were coded. Variables are converted to dimensionless coded values, according to Equation (2) [20].

(2) $$\;x_{i}=\frac{\left(X_{i}-X_{0}\right)}{\Delta X_{i}}\;$$

where *x_i_* represents a coded value of the variable (−1, 0, +1), *X_i_* represents the actual value of the variable, *X*_0_ represents the real value of a variable at the center point, and Δ*X_i_* represents the step change value. The sintering temperature (*X*_1_), the cold pressure (*X*_2_), and the holding time (*X*_3_) were the three variables that were selected. As shown in Table 2, all variables had three levels. The three-factor-three-level design was used for the quadratic response surface and the second-order polynomial model. Therefore, 17 experiments that include 12 factorial runs with five repetitive runs at the central point for estimation of the pure error sum of squares were employed. Table 3 shows the range of variables and the experimental design. The response value can be predicted by the quadratic polynomial Equation (3) [20,21].

(3) $$\;Y=\beta_{0}+\sum \beta_{i}x_{i}+\sum \beta_{ii}x_{i}^{2}+\sum \beta_{ij}x_{i}x_{j}+\varepsilon \;$$

where *Y* represents the predicted value *β*_0_, *β_i_*, *β_ii_*, and *β_ij_* are invariant regression coefficients of the model, *x_i_* and *x_j_* (i=1→3;j=1→3;i≠j) represent the independent variables in the form of coded values, and ε are random errors. Experiments were carried out according to the experimental design shown in Table 3. Design-Expert. 8.0.6 Software (STAT-EASE Inc., Minneapolis, MN, USA) was used to process and analyze experimental data.

## 4. Results and Discussion

### 4.1. ANOVA Analysis and the Adequacy of the BBD Model

Analysis of variance was used to statistically test the response model. Design-Expert software was used for the analysis of variance. Parameters including F-test, P-value, and R-squared are used to analyze the variance, which give an appropriateness and importance to the model.

Table 4, Table 5 and Table 6 show the results of ANOVA analysis for relative density, flexural strength, and the abrasive ratio of the diamond tool bits, respectively. A model with a good ability to fit the data is required to have the highest R-squared value (R^2^) and adjusted R-squared (R^2^-adj). This means that the closer the value of R^2^ is equal to 1, the more accurately the model can describe the relationship between the variables and the response variables. Generally, R^2^ should be required to be at least 0.8 for the models with well-fitted data [22]. In this work, the determining factors of the model for relative density, flexural strength, and abrasive ratio are 0.9998, 0.9960, and 0.9942, respectively. This proves that the model accurately correlates the experimental data for three parameters. Specifically, in Table 4, the predicted R-squared value of 0.9970 is reasonably consistent with the adjusted R-squared of 0.9994. This showed that the predicted data was consistent with the experimental data. The model F value of 3113.22 means that the model was significant while the values of prob > F, which is less than 0.05, indicated that the model terms were significant. In this case, the terms of *X*_1_, *X*_3_, *X*_1_*X*_3_, *X*_1_^2^, and *X*_2_^2^ were significant and played an important role in controlling the relative density of diamond tool bits. The consistency of the BBD model was verified by a lack of fit testing of experimental data. The F-value of the lack of fit is 3.68 (>0.05). This means that it was not significant in the response due to a pure error. In addition, this showed the adequacy of the model [23]. The signal to noise ratio measured by Adeq Precision needs to be greater than 4. The ratio of 135.555 indicated that there are enough signals in this model. From Table 5, the predicted R-squared value of 0.9508 is reasonably consistent with the adjusted R-squared value of 0.9908. This showed that the predicted data was consistent with the experimental data. The model F value of 191.99 means that the model was significant while the values of prob > F less than 0.05 indicated that the model terms were significant. In this case, the terms of *X*_1_, *X*_1_^2^, and *X*_2_^2^ were significant and played an important role in controlling the flexural strength of diamond tool bits. The consistency of the BBD model was verified by a lack of fit testing of experimental data. The F-value of the lack of fit is 3.72 (>0.05). This means that it was not significant in the response due to pure error. In addition, it showed the adequacy of the model. The signal to noise ratio measured by the Adeq Precision tools needs to be greater than 4. The ratio of 33.938 indicated that there are enough signals in this model. As far as Table 6 is concerned, the predicted R-squared value of 0.9425 is reasonably consistent with the adjusted R-squared value of 0.9867. This showed that the predicted data was consistent with the experimental data. The model F value of 133.02 means that the model was significant while the values of prob > F less than 0.05 indicated that the model terms were significant. In this case, the terms of *X*_1_, *X*_2_, *X*_1_*X*_2_, *X*_2_*X*_3_, *X*_1_^2^, and *X*_2_^2^ were significant and played an important role in controlling the flexural strength of diamond tool bits. A lack of fit test was performed on the experimental data to verify the consistency of the BBD model. The F-value of the lack of fit is 1.81 (>0.05), which indicated that it was not significant in responses due to the pure error and showed the adequacy of the model. The signal-to-noise ratio measured by Adeq Precision tools needs to be greater than 4. The ratio of 34.335 indicated that there are enough signals in this model.

**Figure 2:**
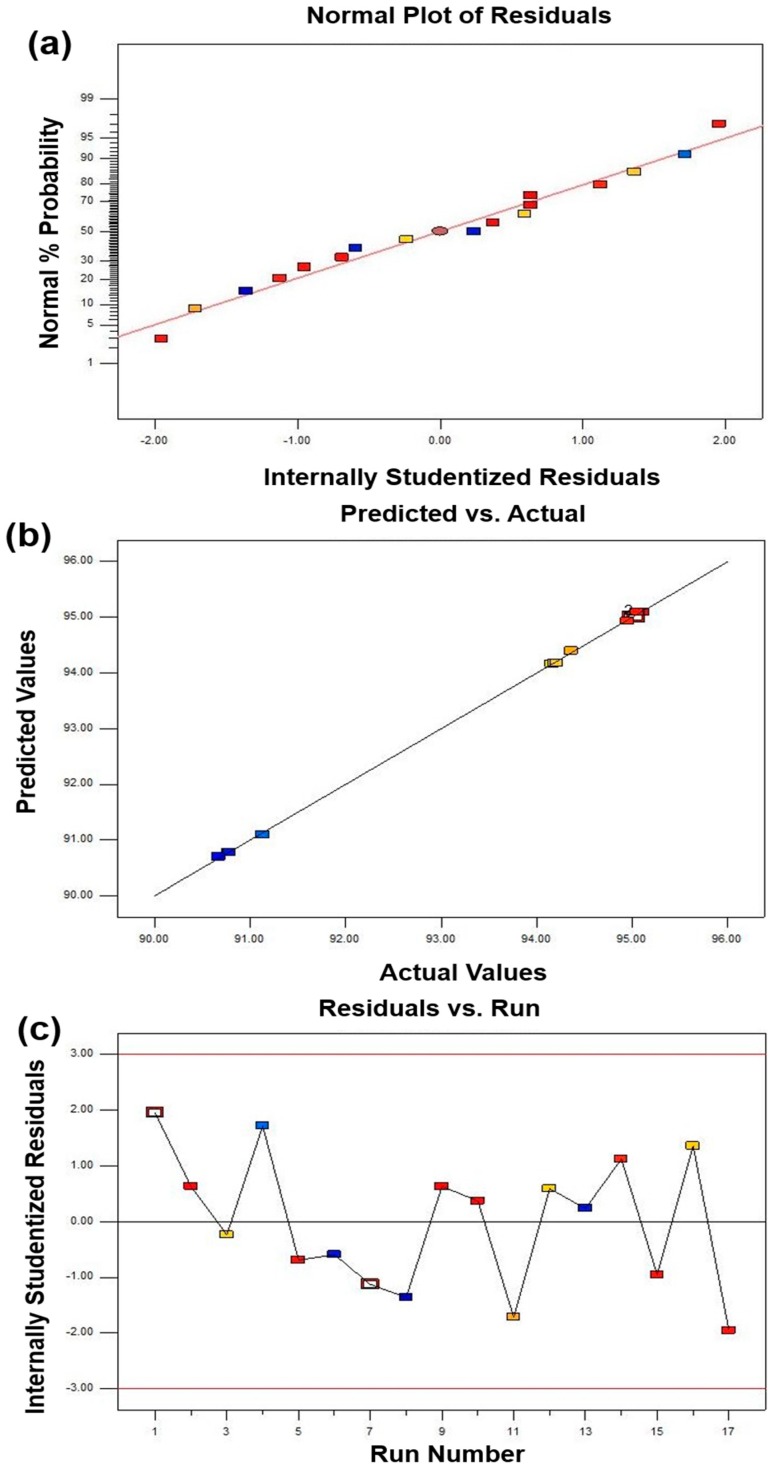
The adequacy of diagnostic maps of the relative density BBD model: (**a**) comparison of normal probability with internal studentized residuals, (**b**) comparison between actual and predicted values, and (**c**) internally studentized of each experimental point.

**Figure 3:**
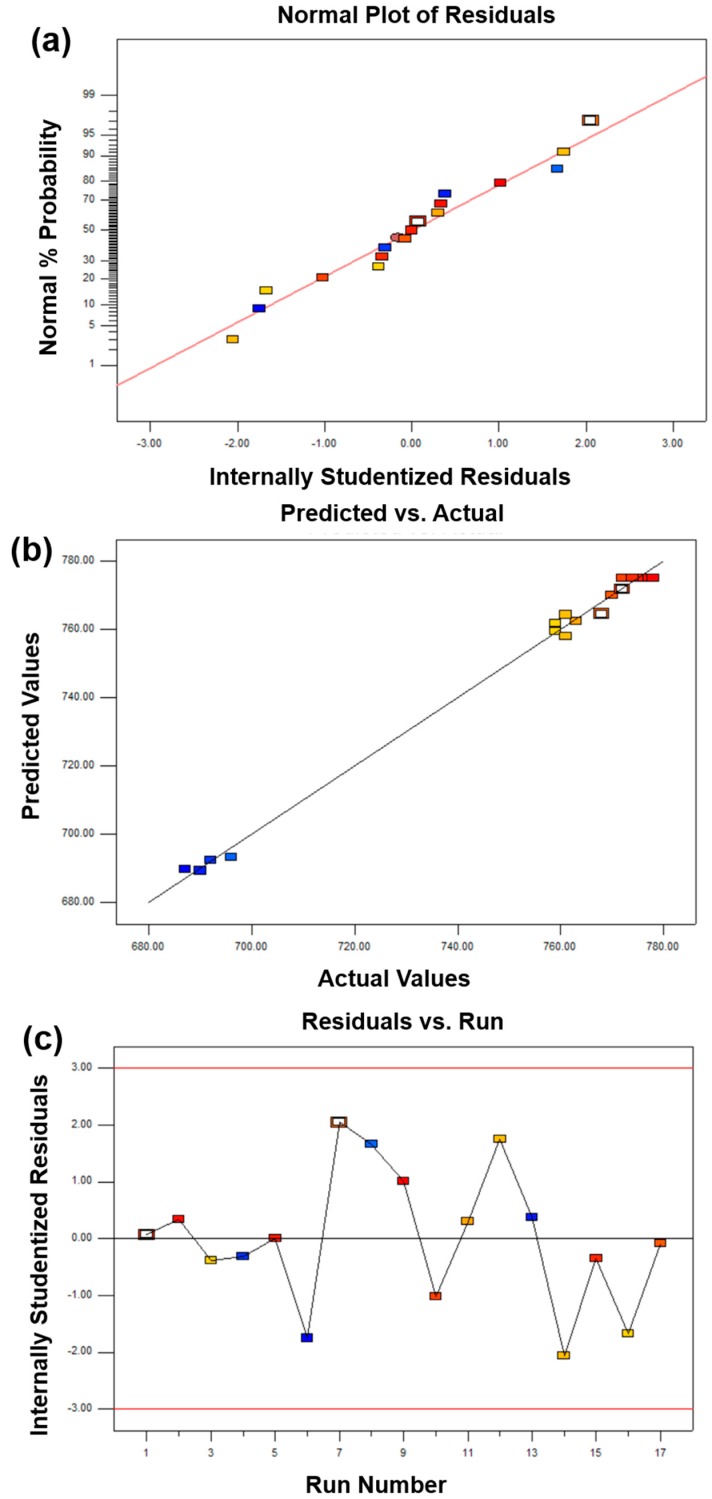
The adequacy of diagnostic maps of the flexural strength BBD model: (**a**) comparison of normal probability with internal studentized residuals, (**b**) comparison between actual and predicted values, and (**c**) internally studentized of each experimental point.

**Figure 4:**
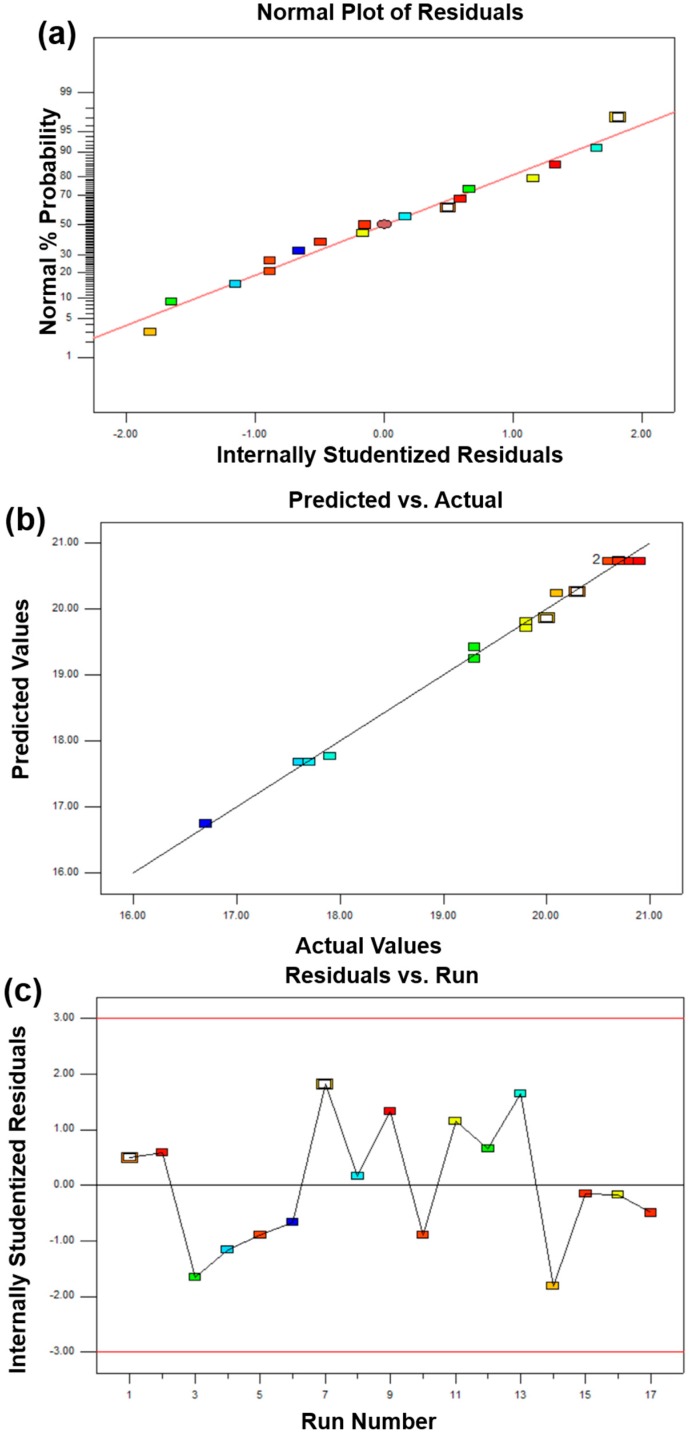
The adequacy of diagnostic maps of the abrasive ratio BBD model: (**a**) comparison of normal probability with internal studentized residuals, (**b**) comparison between actual and predicted values, and (**c**) internally studentized of each experimental point.

The above analysis indicates that the BBD model can be suitable for this work. However, poor or misleading results might be generated for fitting the response surface model. Hence, it is necessary to check the adequacy of the model [17]. The adequacy of the model was checked through various diagnoses such as a normal percentage probability, internally studentized residuals, and predicted versus actual values (Figure 2, Figure 3 and Figure 4). A normal percentage probability plot of residuals represented the normal distribution of the residuals for a response. A normal percentage probability plot is normally distributed and no large variance deviations occurred due to the data points on the graph, which are reasonably close to the line (Figure 2a, Figure 3a and Figure 4a). Figure 2b, Figure 3b and Figure 4b showed the relationship between the predicted and actual values obtained from the BBD model. The predicted value is very close to the experimental value, which indicates that the predicted value is fully consistent with the actual data. The satisfactory fit of the model was analyzed by constructing the relative relationship between internally studentized residuals and experimental runs of 17 experimental results (Figure 2c, Figure 3c and Figure 4c). The smaller the absolute value of the ordinate, the more reliable the experimental data. In this work, all experimental data lie within the limits (−3 to +3). More specifically, the vast majority of the data is in the range of −2 to +2, which is convicted at the 95% confidence level. Therefore, these results indicated that the prediction of the model was accurate.

In summary, based on the analysis of Table 4, Table 5 and Table 6 and Figure 2, Figure 3 and Figure 4, RSM with a BBD model can be used to guide the design and the preparation of diamond tool bits by MPS with appropriate mechanical properties.

### 4.2. The Proposed Fitted Model and Optimization of Microwave Sintering Conditions

Based on the above analysis, the optimization of the MPS by RSM with the BBD model should be accurate. Furthermore, a regression model was established by a second order polynomial equation to study the optimal MPS conditions that could predict the optimal mechanical properties of sintered samples and the combined relationships between the parameters and the response value. The second order polynomial equation obtained through fitting is Equations (4)–(6).

(4) $$\;Y_{R}=95.09+1.70x_{1}+1.25\times 10^{-3}x_{2}-0.045x_{3}+5.00\times 10^{-3}x_{1}x_{2}+0.15x_{1}x_{3}\;-0.038x_{2}x_{3}-2.51\times 10^{-3}x_{1}^{2}-0.098x_{2}^{2}+0.02x_{3}^{2}\;$$

(5) $$\;Y_{F}=775.00+34.63x_{1}-0.50x_{2}+0.37x_{3}-0.25x_{1}x_{2}-3.25x_{2}x_{3}\;-45.50x_{1}^{2}-5.25x_{2}^{2}-2.00x_{3}^{2}\;$$

(6) $$\;Y_{A}=20.72+1.04x_{1}+0.21x_{2}-0.025x_{3}-0.30x_{1}x_{2}-0.025x_{1}x_{3}-0.22x_{2}x_{3}\;-1.99x_{1}^{2}-0.44x_{2}^{2}-1.00\times 10^{-2}x_{3}^{2}\;$$

**Figure 5:**
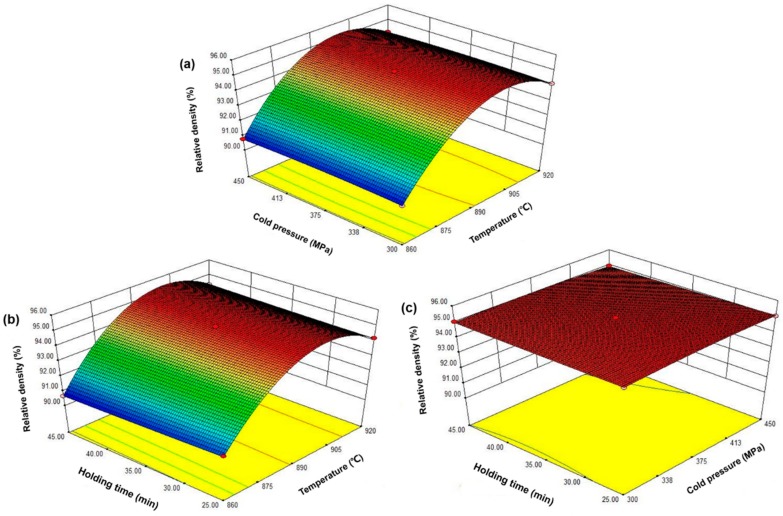
Response surface graph: the effect of sintering temperature, holding time, and cold pressure on the relative density of the sintered sample, (**a**) sintering temperature and holding time; (**b**) sintering temperature and cold pressure; (**c**) holding time and cold pressure.

**Figure 6:**
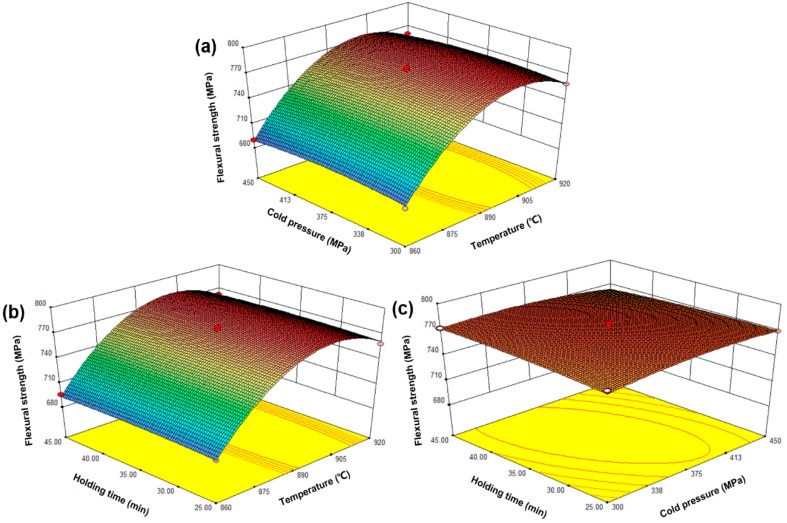
The response surface graph: the effect of sintering temperature, holding time, and cold pressure on the flexural strength of the sintered sample, (**a**) sintering temperature and holding time; (**b**) sintering temperature and cold pressure; (**c**) holding time and cold pressure.

**Figure 7:**
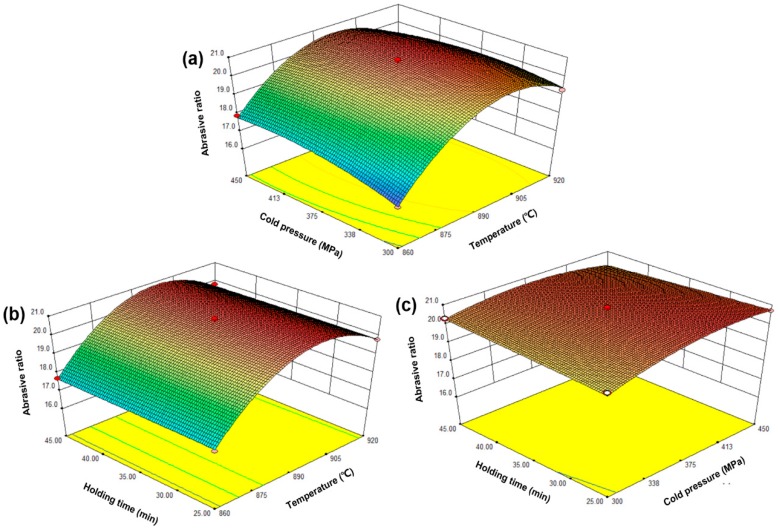
The response surface graph: the effect of sintering temperature, holding time, and cold pressure on the abrasive ratio of the sintered sample, (**a**) sintering temperature and holding time; (**b**) sintering temperature and cold pressure; (**c**) holding time and cold pressure.

The purpose of the optimization was to study different MPS conditions, which could obtain better mechanical properties of the sintered samples. Equations (4)–(6) show that the factor coefficient named *x*_1_ is larger than other factor coefficients, which means that changing the factor *x*_1_ has more effect on relative density, flexural strength, and the abrasive ratio than other factors. Therefore, the sintering temperature is more effective than the cold pressure and the holding time. Intuitively, Figure 5 shows the effect of sintering temperature, holding time, and cold pressure on the relative density of the sintered samples. As seen from the figure, the relative density of the sintered samples was significantly affected by the sintering temperature. Specifically, the density of the sintered sample increase significantly with temperatures up to 900 °C and slightly decreased after exceeding 900 °C. The holding time and the cold pressure have little effect on the relative density of the sintered sample. Figure 6 shows the effect of the sintering temperature, the holding time, and the cold pressure on the flexural strength of the sintered samples. As seen from the figure, the trend of change is similar to that of Figure 5. However, compared with Figure 5, the influence of the cold pressure on the bending strength of the sintered sample increases. Similarly, Figure 7 represents the effect of sintering time, the holding time, and the cold pressure on the abrasive ratio of the sintered product. Temperature plays a major role and the effect of the cold pressing pressure is also increased. Clearly, the sintering temperature is a major factor affecting the relative density, the flexural strength, and the abrasive ratio of the sintered samples. There are two possible reasons for this. On the one hand, a very high sintering temperature leads to the loss of liquid phase elements (mainly Sn). On the other hand, an excessively high sintering temperature causes the internal crystal grain moving speed of the sample to increase and exceed the gas discharge speed, which hinders the gas discharge and causes an increase in the internal pores of the sample [24]. The effect of cold pressure on the flexural strength and abrasive ratio of the sintered sample is more clear than the effect on the relative density. The possible reason for this is that increasing the cold pressure allows the metal matrix to have a better holding effect on the diamond. In addition, increasing the cold pressure can also make the green compact sample denser while also reducing the sample’s ability to respond to microwaves [25]. The holding time has no significant effect on the relative density, the flexural strength, and the abrasive ratio of the sintered samples. The possible reason for this is that the sintering process can already be completed within the selected holding time range. The excessive holding time may coarsen the internal crystal grains of the sample and lower the mechanical properties [26]. Furthermore, the optimized MPS conditions of the sintered sample by used Design-Expert. 8.0.6 Software. The optimized sintering conditions were sintering temperature of 900 °C, the cold pressure of 395 MPa, and the holding time of 30 min. In order to confirm the predicted results of the model, repeated experiments were performed under optimal conditions. In addition, the percent of the absolute relative error (ARE%) is used to re-estimate the accuracy of the model below [22].

(7) $$\;AR_{E}\%=\frac{\left|X_{i,exp-}X_{i,pre}\right|}{X_{i,exp}}\;$$

The experimental results are shown in Table 7. The average values of the relative density, the flexural strength, and the abrasive ratio of the sintered sample obtained by the experiment were 95.17%, 775.67 MPa, and 20.4, respectively, and the predicted values were 95.38%, 780 MPa, and 20.9. It can be calculated that the ARE% of the relative density, the flexural strength, and the abrasive ratio are only 0.22%, 0.56%, and 2.4%, respectively. This indicates that the experimental values are quite close to the predicted values and there is no significant difference, which proves the validity of the optimization conditions of the BBD model.

### 4.3. Comparison of Conventional and Microwave Sintering

The optimized process conditions for MPS were obtained based on the above studies. Whether MPS is superior to CPS. For this purpose, CPS experiments were carried out under the same sintering conditions (sintering temperature of 900 °C, the cold pressure of 395 MPa, and the holding time of 30 min). The experimental results are shown in Table 8. The average values of the relative density, the flexural strength, and the abrasive ratio of the sintered sample obtained by the experiment were 89.25%, 605 MPa, and 15.2, respectively. It can be seen from Table 7 and Table 8, when compared with CPS, the relative density, the flexural strength, and the abrasive ratio of the sintered sample after MPS are increased by 6.75%, 20.3%, and 22.2%, respectively. The possible reason for this is that there is a fundamental difference between microwave heating and conventional heating. For the CPS method, the direction of heat conduction in the sample is from the outside to the inside, which is opposite to the direction in which the gas escapes during densification. Therefore, the contradictory direction is not conducive to gas emissions and shrinkage of the microstructures. Furthermore, under pressureless conditions, the densification of the metal matrix depends on the diffusion filling of the liquid phase elements and the alloying process between the metal atoms. The clear difference is that the microwave energy is directly absorbed by the metal powder during the MPS and the in situ energy conversion occurs through the dielectric loss and the magnetic conduction loss of the raw material. This heating mode embodies the characteristics of internal enhanced heating. The direction of the temperature gradient is consistent with the direction of gas discharge, which is conducive to gas discharge and reduces the microstructural defects of the sample. In addition, the in situ conversion of microwave energy into kinetic energy and the potential energy of metal atoms can reduce the activation energy of internal reactions and promote the alloying reaction of the metal to make the metal matrix and diamond more tightly combined [13,27].

**Figure 8:**
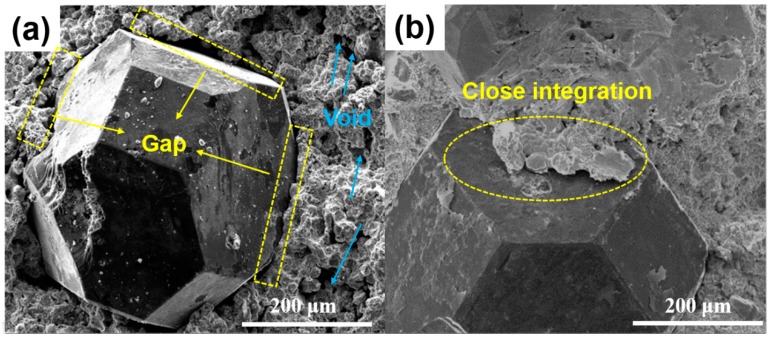
SEM pictures of microstructure morphology of samples sintered by (**a**) CPS (900 °C, 395 MPa, 30 min), (**b**) MPS (900 °C, 395 MPa, 30 min).

**Figure 9:**
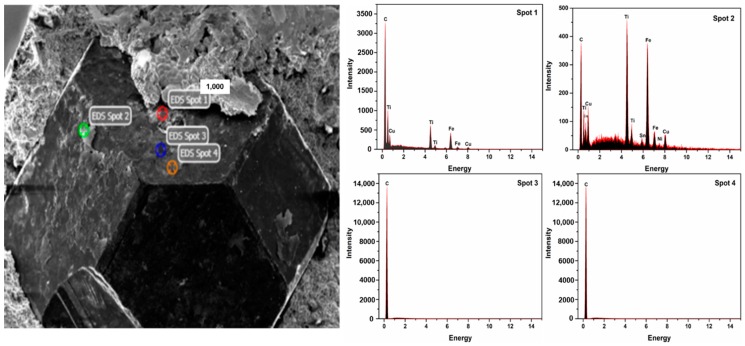
SEM-EDX analysis of samples sintered by MPS (900 °C, 395 MPa, 30 min).

**Figure 10:**
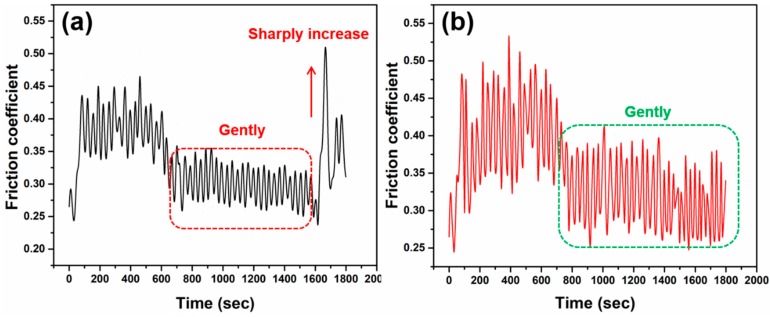
Variation of the friction coefficient of samples sintered by (**a**) CPS (900 °C, 395 MPa, 30 min). (**b**) MPS (900 °C, 395 MPa, 30 min).

The microstructure of sintered samples was studied by SEM and EDX (Figure 8 and Figure 9). Figure 8a represents a conventional sintered sample and it can be seen that there are many voids and the diamond has a clear gap with the metal matrix and the crystal grains are coarse. These reasons lead to poor mechanical properties such as relative density, flexural strength, and an abrasive ratio. Compared with the microwave sintered samples (Figure 8b), the diamond and the metal matrix are closely integrated, the pores are small, and the crystal grains are fine, which is beneficial for improving the mechanical properties of the sample. Furthermore, it can be seen from Figure 9 that the close integration of the diamond and the metal matrix is mainly due to the combination of Ti and diamond. It is apparent that Ti aggregation occurs at spot 1 and 2 in Figure 9. The possible reason for this is that the in situ conversion of microwave energy promotes the metallurgical bonding of Ti and diamonds [27,28]. Figure 10 shows the variation of the friction coefficient of the sintered sample during the test of the abrasive ratio. The coefficient of friction can be used to characterize the stability of the sample during grinding. The friction coefficient of conventional sintered samples sharply increases at 1600 s (Figure 10a). This indicates that the diamond in the sample has abnormal shedding and reduces the working efficiency. In contrast, microwave sintered samples are more stable (Figure 10b), which is more efficient for diamond tools. In general, the condition of the sample microstructure reflects the consistency with Table 7 and Table 8.

### 4.4. The Possible Mechanism of MPS

**Figure 11:**
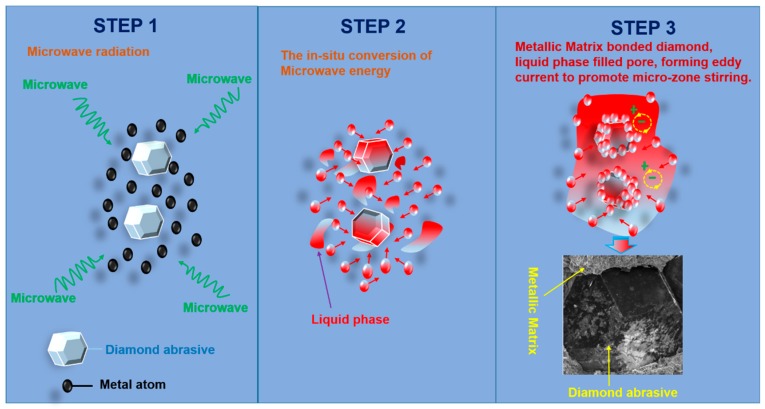
The schematic diagram of a possible mechanism for the MPS.

Previous work has shown that the mechanical properties of the metal matrix was sintered by the MPS method better than those obtained by the CPS method [7]. This may be due to different heating principles of microwaves and conventional sintering. In this work, diamond and metal powder are mixed and then sintered. Therefore, the interactions between diamonds and metal powder should be considered. We propose a possible MPS mechanism, which is shown in Figure 11. The absorption of microwave energy by metal atoms and its in situ conversion into thermal energy enhances atomic motion, which facilitates the combination of metal atoms and metal atoms with diamonds [29,30]. At the same time, Sn becomes a liquid phase to form a migration mechanism and excludes gas. In addition, there may be magnetic effects in the microwave field that result in the formation of micro-zone electromagnetic stirring to refine the grains in order to enhance mechanical properties [31,32]. Figure 11 demonstrates the variation process during microwave sintering. It should be pointed out that the advantages of microwave sintering have been reflected, but the in-depth sintering mechanism remains to be explored. In this work, we only propose possible microwave sintering mechanisms.

## 5. Conclusions

The metallic matrix diamond tool bits were successfully fabricated by MPS. The BBD response surface experiment design was used to optimize the sintering process parameters. From the experimental design, a regressive quadratic mathematical model was developed and applied to optimize the preparation parameters to obtain the maximum mechanical properties of the sintered sample. After doing some trial experiments and real experiments, it was found that a sintering temperature of 900 °C, a cold pressure of 395 MPa, and a holding time of 30 min are the optimal preparation conditions. The average values of the relative density, the flexural strength, and the abrasive ratio of the sintered sample obtained by the experiment were 95.17%, 775.67 MPa, and 20.4, respectively. When comparing with CPS, the relative density, the flexural strength, and the abrasive ratio of the sintered sample after MPS were increased by 6.75%, 20.3%, and 22.2%, respectively. Lastly, it was uncovered that the MPS method for the preparation of metal-based diamond tools is a potential method with a real-time process. Applications of MPS technology for the preparation of metallic matrix diamond tools are now in hand.

## Figures and Tables

**Table 1:** Content of raw materials.

Ingredient	Cu	Fe	Co	Sn	Ni	Ti	Diamond
Content (wt.%)	40	25	13	7	8	2	5

**Table 2:** The actual value and coding of the variables.

Variable *X*	Levels		
	Low (−1)	Center (0)	High (1)
*X*_1_: Sintering temperature (°C)	860	890	920
*X*_2_: Cold pressure (MPa)	300	375	450
*X*_3_: Holding time (min)	25	35	45

**Table 3:** Experimental design and results of BBD.

Run	Variable			Response Variable					
	*X*_1_	*X*_2_	*X*_3_	Relative Density (%)		Flexural Strength (MPa)		Abrasive Ratio	
				Predicted	Actual	Predicted	Actual	Predicted	Actual
1	890	300	45	95.00	95.04	772	772	20.3	20.3
2	890	375	35	95.09	95.11	775	776	20.7	20.8
3	920	300	35	94.16	94.16	760	759	19.4	19.3
4	860	375	25	91.09	91.13	693	692	17.7	17.6
5	890	375	35	95.09	95.06	775	775	20.7	20.6
6	860	300	35	90.78	90.77	689	687	16.8	16.7
7	890	300	25	95.01	94.99	765	768	19.9	20.0
8	860	375	45	90.70	90.67	694	696	17.7	17.7
9	890	375	35	95.09	95.11	775	778	20.7	20.9
10	890	375	35	95.09	95.10	775	772	20.7	20.6
11	920	375	45	94.40	94.36	763	763	19.7	19.8
12	920	450	35	94.18	94.19	758	761	19.3	19.3
13	860	450	35	90.78	90.78	689	690	17.8	17.9
14	890	450	45	94.93	94.95	764	761	20.2	20.1
15	890	375	35	95.09	95.05	775	774	20.7	20.7
16	920	375	25	94.18	94.21	762	759	19.8	19.8
17	890	450	25	95.09	95.05	770	770	20.7	20.6

**Table 4:** Analysis of variance (ANOVA) of relative density for selected experimental correlation using RSM.

Source	Sum of Squares	Degrees of Freedom	Mean Square	F-Value	P-Value Prob > F	
Model	50.01	9	5.56	3113.22	<0.0001	Significant
*X*_1_	23.02	1	23.02	12,895.30	<0.0001	
*X*_2_	1.25 × 10^−5^	1	1.25 × 10^−5^	7.00 × 10^−3^	0.9357	
*X*_3_	0.0162	1	0.02	9.08	0.0196	
*X*_1_ *X*_2_	1.00 × 10^−4^	1	1.00 × 10^−4^	0.06	0.8197	
*X*_1_ *X*_3_	0.09	1	0.09	52.11	0.0002	
*X*_2_ *X*_3_	0.01	1	0.01	3.15	0.1191	
*X*_1_^2^	26.59	1	26.59	14,896.46	<0.0001	
*X*_2_^2^	0.04	1	0.04	22.65	0.0021	
*X*_3_^2^	1.60 × 10^−3^	1	1.60 × 10^−3^	0.90	0.3751	
Residual	0.01	7	1.79 × 10^−3^			
Lack of fit	0.01	3	3.06 × 10^−3^	3.68	0.1200	Not significant
Pure error	3.32 × 10^−3^	4	8.30 × 10^−4^			
Corrected total	50.03	16				

R-Squared: 0.9998, Pred R-Squared: 0.9970, Adj R-Squared: 0.9994, Adeq Precision: 135.555.

**Table 5:** Analysis of variance (ANOVA) of flexural strength for selected experimental correlation using RSM.

Source	Sum of Squares	Degrees of Freedom	Mean Square	F-Value	P-Value Prob > F	
Model	18,698.72	9	2077.64	191.99	<0.0001	Significant
*X*_1_	9591.13	1	9591.13	886.31	<0.0001	
*X*_2_	2.00	1	2.00	0.18	0.6802	
*X*_3_	1.13	1	1.13	0.10	0.7565	
*X*_1_ *X*_2_	0.25	1	0.25	0.02	0.8835	
*X*_1_ *X*_3_	0.00	1	0.00	0.00	1.0000	
*X*_2_ *X*_3_	42.25	1	42.25	3.90	0.0887	
*X*_1_^2^	8716.84	1	8716.84	805.52	<0.0001	
*X*_2_^2^	116.05	1	116.05	10.72	0.0136	
*X*_3_^2^	16.84	1	16.84	1.56	0.2523	
Residual	75.75	7	10.82			
Lack of fit	55.75	3	18.58	3.72	0.1186	Not significant
Pure error	20.00	4	5.00			
Corrected total	18,774.47	16				

R-Squared: 0.9960, Pred R-Squared: 0.9508, Adj R-Squared: 0.9908, Adeq Precision: 33.938.

**Table 6:** Analysis of variance (ANOVA) of the abrasive ratio for the selected experimental correlation using RSM.

Source	Sum of Squares	Degrees of Freedom	Mean Square	F-Value	P-Value Prob > F	
Model	27.45	9	3.05	133.02	<0.0001	Significant
*X*_1_	8.61	1	8.61	375.57	<0.0001	
*X*_2_	0.36	1	0.36	15.76	0.0054	
*X*_3_	0.00	1	0.00	0.22	0.6547	
*X*_1_ *X*_2_	0.36	1	0.36	15.70	0.0054	
*X*_1_ *X*_3_	0.00	1	0.00	0.11	0.7509	
*X*_2_ *X*_3_	0.20	1	0.20	8.83	0.0208	
*X*_1_^2^	16.59	1	16.59	723.57	<0.0001	
*X*_2_^2^	0.80	1	0.80	34.75	0.0006	
*X*_3_^2^	0.00	1	0.00	0.02	0.8960	
Residual	0.16	7	0.02			
Lack of fit	0.09	3	0.03	1.81	0.2844	Not significant
Pure error	0.07	4	0.02			
Corrected total	27.61	16				

R-Squared: 0.9942, Pred R-Squared: 0.94.25, Adj R-Squared: 0.9867, Adeq Precision: 34.335.

**Table 7:** Optimal condition values of microwave sintering conditions.

Optimum Results	*X_1_*: Temperature (°C)	*X_2_*: Cold Pressure (MPa)	*X_3_*: Holding Time (min)	Experimental Value		Predicted Value	ARE%
					Average		
Relative density (%)	900	395	30	95.26	95.17	95.38	0.22
				94.95			
				95.29			
Flexural strength (MPa)	900	395	30	774	775.67	780	0.56
				772			
				779			
Abrasive ratio	900	395	30	20.5	20.4	20.9	2.4
				20.1			
				20.7			

**Table 8:** Experimental results of conventional sintering.

Indexes	Temperature (°C)	Cold Pressure (MPa)	Holding Time (min)	Experimental Value	
					Average
Relative density (%)	900	395	30	89.15	89.25
				88.97	
				89.62	
Flexural strength (MPa)	900	395	30	645	646
				636	
				657	
Abrasive ratio	900	395	30	16.2	16.7
				16.7	
				17.3	
